# Testing Measurement and Factor Structure Invariance of the Physical Activity and Leisure Motivation Scale for Youth Across Gender

**DOI:** 10.3389/fpsyg.2018.01096

**Published:** 2018-07-03

**Authors:** Yee Cheng Kueh, Nurzulaikha Abdullah, Garry Kuan, Tony Morris, Nyi Nyi Naing

**Affiliations:** ^1^Unit of Biostatistics and Research Methodology, School of Medical Sciences, Universiti Sains Malaysia, Kelantan, Malaysia; ^2^Exercise and Sports Science, School of Health Sciences, Universiti Sains Malaysia, Kelantan, Malaysia; ^3^Institute of Sport, Health and Active Living, College of Sport and Exercise Science, Victoria University, Melbourne, VIC, Australia; ^4^Institute for Community (Health) Development (i-CODE), Universiti Sultan Zainal Abidin, Terengganu, Malaysia

**Keywords:** physical activity, motivation, gender, confirmatory factor analysis, invariance

## Abstract

Measurement equivalence is often assumed across comparison groups, a pervasive problem related to many self-report instruments. Measurement equivalence, also known as measurement invariance, implies that a measure has the same meaning across different groups of people. In this study, we aimed to examine the measurement and structural invariance among gender of the Malay version of the Physical Activity and Leisure Motivation Scale for Youth (PALMS-Y-M). Seven-hundred-and-eighty-three secondary school students (female = 57.3%, male = 42.7%) with mean age 14.5 years (standard deviation = 1.25) from Kota Bharu, Malaysia, volunteered to participate in this study and completed the PALMS-Y-M, consisting of 28 items with seven subscales. We conducted the confirmatory factor analysis (CFA) and invariance tests on the seven motives of the PALMS-Y-M model. The hypothesized model consisted of 28 observed items and seven latent variables. We used estimator robust to maximum likelihood, MLR to examine the hypothesized measurement and structural invariance. Measurement invariance was tested for three different levels. We first established the configural invariance model, then we compared the metric invariance model and the scalar invariance model with the less restrictive model. Then structural invariance was tested for factor variance, covariance, and means. Findings provided evidence for full measurement and structural invariance of the PALMS-Y-M in males and females. The final CFA model fit the data well for males [comparative fit index (CFI) = 0.922, root mean square error of approximation (RMSEA) = 0.048, standardized root mean residual (SRMR) = 0.050] and females (CFI = 0.922, RMSEA = 0.047, SRMR = 0.053). When invariance of both factor loadings and item intercepts holds in PALMS-Y-M, underlying factors consisting of different motives for participating in PA can be meaningfully compared across gender. Accurate and valid measurement of PALMS-Y-M across comparison groups is crucial for future research that involves examining motives to physical activity in different genders and other socio-cultural variables.

## Introduction

Regular physical activity is important to maintain physical health, fight against obesity, and treat a variety of chronic health conditions. Lack of physical activity causes obesity and complications due to chronic illness, such as coronary heart disease, colon cancer, hypertension, and diabetes (U. S. Department of Health and Human Services, 2008). Moreover, researchers have identified the association between inactivity and different types of cancer, such as breast cancer, and cancer of the colon, endometrium, esophagus, kidney, pancreas, and cervix (Kushi et al., 2006; Lee et al., 2012). Conversely, regular physical activity could contribute to increased mental health and academic performance, lower stress, and depression levels (Nieman, 2002; Rasberry et al., 2011), decrease the risk of coronary heart disease or cardiovascular disease (Williams, 2001), and prolong life expectancy (Lee et al., 2012).

Lack of physical activity among youth has become a major concern in societies. Nearly half of American young people aged between 12 and 21 are not vigorously physical active on a regular basis (U. S. Department of Health and Human Services, 2008). Individuals who were physically inactive during adolescence are also more likely to be inactive in their adulthood (Gordon-Larsen et al., 2004). Physical activity during youth can help protect against many types of health risks in adulthood. Studies have shown that adolescents who actively engage in physical activity, such as aerobic exercise, demonstrated improvement in math skills, cognitive flexibility, improved memory, and creativity (Hillman et al., 2008; Chaddock et al., 2011; Davis et al., 2011). Therefore, youth should be encouraged and motivated to engage in physical activity regularly because there are many immediate benefits for their health.

Motivation has become a key factor in maintaining individuals’ physical activity. Research had demonstrated the link between motivation and physical activity (Frederick et al., 1996; Kilpatrick et al., 2005; Wilson et al., 2008; Eisenberg, 2014; Slovinec et al., 2014). Individuals who are intrinsically motivated to undertake physical activity are motivated by factors, including enjoyment, challenge, skill development, and mastery (Frederick and Ryan, 1993; Kilpatrick et al., 2005), whereas individuals who are extrinsically motivated to undertake physical activity are motivated by factors that are not related to the activity itself, including rewards, improved health, and appearance (Frederick and Ryan, 1993; Kilpatrick et al., 2005). Therefore, understanding people’s motives for participation in physical activity is crucial given its role in determining whether individuals will initiate and maintain physical activity programs. Physical inactivity is evident in both genders, all socioeconomic, and cultural categories, and at all ages (Weinberg et al., 2000; Molanorouzi et al., 2015). Nonetheless, the low and reducing prevalence of physical activity in younger people is of particular concern (Chaddock et al., 2011; Hu et al., 2015; Abdullah, 2016). More research should be conducted to identify the motives of youth participating in and abstaining from physical activity in order to alter the increasing trend of physical inactivity among youth in many countries.

Reliable and valid measures are important to identify adolescents’ motives for participating in any physical activity. Several questionnaires have been developed to measure motivation for physical activity participation, including the Sport Motivation Scale (SMS; Fortier et al., 1995), the Exercise Motivation Inventory (EMI; Markland and Ingledew, 1997), the Exercise Motivation Scale (EMS; Li, 1999), the Motivation for Physical Activity Measure (MPAM; Frederick and Ryan, 1993), the Recreational Exercise Motivation Measure (REMM; Rogers and Morris, 2003), and the Physical Activity and Leisure Motivation Scale (PALMS; Morris and Rogers, 2004). PALMS is the revised and shortened version of the 73-item REMM. It consists of 40 items that provide information about individuals’ motives for participation in physical activity. There are eight domains measured in PALMS: mastery, enjoyment, psychological condition, physical condition, appearance, others’ expectations, affiliation, and competition/ego. These questionnaires (i.e., SMS, EMI, EMS, MPAM, REMM, and PALMS) were not specifically developed and used to measure motives for participating in physical activity among adolescents and youth. Therefore, a version of PALMS was developed to measure motives for participation in physical and leisure activity among youth. This was stimulated by observations that younger respondents to PALMS had problems responding to items measuring the motive others’ expectations. Items on this motive refer to motives associated with being paid to participate and participating to address chronic medical conditions neither of which apply to most adolescents. The others’ expectations motive subscale was removed from PALMS. Hu et al. (2015) conducted a study among Chinese schoolchildren using the 35-item, 7-motives version of PALMS that remained, after checking the scale for comprehensibility among 12–15 years old. The model that Hu et al. (2015) tested was sound, but they removed the least strong item from each motive subscale to produce a shorter questionnaire that they considered to be more suitable for use with this age group. Hu et al. (2015) named the resulting 28-item, 7-motive subscale instrument the PALMS-Youth (PALMS-Y). The “parent” measure, REMM, and the shorter version, PALMS, have been validated in Malay language with adult Malaysia-based samples, and the results indicated sound validity and reliability (Kueh et al., 2017a,b). The validity of PALMS based on confirmatory factor analysis (CFA) was satisfactory with fit indices of RMSEA = 0.041, SRMR = 0.052 and the composite reliabilities for the motive subscales were acceptable, ranging from 0.67 to 0.85 (Kueh et al., 2017a). Since the PALMS-Y was developed by selecting the strongest items in the PALMS that are relevant to the youth population, we propose that the PALMS-Y will be reliable and valid. Moreover, the simplicity of the items and their meanings are dedicated to measure levels of motives related to physical and leisure activity. Thus, PALMS-Y is suitable to be used for adolescents who engage in physical activity and leisure across a wide range of activity.

Although previous research had confirmed the validity of PALMS using CFA, researchers had never tested for equivalence of PALMS and PALMS-Y among different genders. This means it has not been demonstrated that males and females understand the measure in a similar manner. In addition, research on gender differences in motivation indicates that males and females exhibit different primary motives for participation in physical activity (Frederick and Ryan, 1993; Morris et al., 1995; Weinberg et al., 2000). For example, females have consistently rated appearance motives more highly than their male counterparts, whereas males typically score higher on the competition/ego motive than females (Frederick and Ryan, 1993; Frederick and Morrison, 1996; Frederick et al., 1996; Weinberg et al., 2000). It is possible that male and female adolescents would interpret motive items in a motivation questionnaire differently, rather than simply reporting different preferences. For example, the item “to improve body shape” may be interpreted by female as improving body to a slim and possible underweight level that is commonly depicted in entertainment social media, especially in Asian countries, whereas males may interpret improved body shape to be associated with being muscular and strong. In order to make a meaningful comparison between genders, we need to demonstrate that the PALMS-Y is valid and invariant among gender, when measuring the motives of male and female adolescents for participation in physical activity. Measurement invariance is a prerequisite for demonstrating that constructs are comparable across groups (Brown, 2006; Wang and Wang, 2012). Without knowing whether the measure used is invariant across groups of interest, researchers cannot be certain whether observed differences represent genuine differences between those groups or whether they result from differences in interpretation. Thus, it is necessary to examine whether similarities and differences across genders derived, when using PALMS-Y to measure motives, are meaningful.

Until the development of the PALMS-Y and its testing in a Chinese adolescent population, there has been no questionnaire that specifically measures motives for participation in physical and leisure activity specifically in youth. Thus, development and testing the invariance of PALMS-Y has a significant implication for its use in research and health practice in identifying the motives of youth or adolescents for participating in physical and leisure activities. Adolescence is a highly complex transition period from childhood to adulthood. This period is characterized by major biological, physiological, and psychological changes (Lerner et al., 2003), and increased divergence between males and females in physical characteristics, behavior, and brain volume development (Lenroot and Giedd, 2010). Demonstrating measurement invariance of PALMS-Y between males and females, will allow future researchers, health professionals, and physical educators to use this measure confidently irrespective of the gender of adolescents or youth under examination. In the present study, we aimed to validate and confirm the measurement invariance of the Malay version of the PALMS-Y (PALMS-Y-M) among males and females.

## Materials and Methods

### Participants

All participants were Malaysian secondary school students. Participants all spoke, read, and wrote in Malay. The participants’ age ranged from 13 to 17 years. They were enrolled in government-funded secondary schools in Kota Bharu, Malaysia during the data collection period. A total of 783 secondary school students (male 42.7%, *n* = 334, female 57.3%, *n* = 449) participated in this study. The mean age of the participants was 14.5 years [standard deviation (SD) = 1.25], and their ethnicity comprises Malay (57.3%), Chinese (41.3%), Indian (0.3%), and others (1.1%).

### Measures

In the present study, data were collected using a form to gather demographic details and information about physical and leisure activities and the PALMS-Y-M questionnaire.

#### Demographic/Physical and Leisure Activities Information

Several demographic and physical and leisure activity questions were included in a form. These questions included personal attributes of the participants (i.e., age, gender, and ethnicity), the sport or physical and leisure activities participants were involved in, and the duration (in minutes or hours) per week of pursuing the activities.

#### Physical Activity and Leisure Motivation Scale-Youth-Malay (PALMS-Y-M)

The PALMS-Y-M consists of 28 items with seven subscales measuring different type of motives for participating in physical and leisure activities. The seven motives are mastery, enjoyment, psychological condition, physical condition, appearance, affiliation, and competition/ego. Each motive (subscale) of PALMS-Y-M consists of four items. Examples of items and their respective subscales are: to get better at an activity (mastery), because it’s interesting (enjoyment), to better cope with stress (psychological condition), because is helps maintain a healthy body (physical condition), to define muscle, look better (appearance), to do activity with others (affiliation), and because I perform better than others (competition/ego). The 28 items and their respective subscales can be found in Appendix. The response format for all the items is a 5-point Likert scale rated from 1 (*strongly disagree*) to 5 (*strongly agree*), higher scores reflect that participants experience a higher level of that motive for participating in physical and leisure activities. PALMS-Y-M is the short version of Malay version of PALMS (PALMS-M) and PALMS-M has been previously validated in a Malaysia-based sample. In the present sample, the internal consistency for each motive subscale based on Cronbach’s Alpha was as follows: 0.77 (enjoyment), 0.77 (mastery), 0.76 (competition/ego), 0.73 (affiliation), 0.85 (appearance), 0.79 (physical condition), and 0.83 (psychological condition).

### Procedure

Ethics approval for this study was obtained from the Universiti Sains Malaysia Human Research Ethics Committee. Participants and their parents were provided with a research information sheet and written informed consent was obtained from parents prior to the study. All subjects gave written informed consent in accordance with the Declaration of Helsinki. Permission to conduct data collection in schools was also obtained from the Ministry of Education Malaysia, District Office of Education Kota Bharu, and the school principals from the selected secondary schools in Kota Bharu, Kelantan.

A cross-sectional study design was employed in the present study. A cluster sampling method was applied by randomly selecting three out of 48 secondary schools in Kota Bharu, Kelantan. The schools’ principals were contacted for the study. Among the selected secondary schools, all the students who were available during the data collection period were invited to participate in the study. The students were briefed regarding the study and were asked to obtain written consent from their parents or guardians, if they agreed to participate in the study. Those who volunteered to participate in the study completed the demographic form and the PALMS-Y-M questionnaire and returned them to the researcher. A total of 858 questionnaires were distributed to students, and the response rate was 93.2% with 800 questionnaires returned to the researchers. However, 17 questionnaires were not completed fully or appropriately, so there were 783 usable questionnaires with complete answers for data analysis.

### Data Analysis

Data analysis was conducted using Mplus 8 (Muthén and Muthén, 1998–2012). There were no missing data in the final dataset. The responses to all 28 items are ordered from 1 (*strongly disagree*) to 5 (*strongly agree*). The assumption of multivariate normality was not met based on Mardia multivariate skewness and kurtosis tests of fit (*p* < 0.001). Therefore, an alternative estimator robust to maximum likelihood (MLR; Muthén and Muthén, 1998–2012) was used to examine the measurement model.

The hypothesized measurement model consists of seven latent variables (factors) and 28 observed variables (items or indicators). The factor structure of the hypothesized measurement model was tested in CFA. The fit of the measurement model to the data was evaluated by several fit indices. These fit indices were as follows: the comparative fit index (CFI) and the Tucker and Lewis index (TLI), with the desired value of more than 0.92, the root mean square error of approximation (RMSEA), with the desired value of less than 0.07, and the standardized root mean residual (SRMR), with the desired value of less than 0.08 (Hair et al., 2010). The cut off points of the fit indices were taken based on recommendations from Hair et al. (2010) for sample sizes more than 250 and number of observed variables between 12 and 30. The present study on PALMS-Y-M consisted of a sample size of 783 and 28 observed variables, the 28 items of PALMS-Y-M. The Chi-square and its degree of freedom (df) were reported along with other fit indices, although a significant *p*-value can be expected with large sample size (Hair et al., 2010). Factor loadings of 0.40 and above, with significant *p*-values, standardized residuals, and modification indices (MI), were used to locate any problematic items that contributed to misfit to the data (Wang and Wang, 2012). Additional parameters, such as residual covariances among items, were added in the respecification models based on MI values and after consideration of meaningfulness of adding the covariances among the identified items.

Measurement invariance of the scale’s items across gender was tested based on published guidelines for establishing measurement invariance of models (Muthén and Muthén, 1998–2012; Wang and Wang, 2012; Byrne, 2013). We conducted hierarchical tests for invariance of measurement parameters. First, we examined the configured invariance model or pattern invariance, which imposes no equality restrictions on model parameters, including gender in this study. This is a necessary condition for testing invariance by comparing it with other invariance models based on fit indices. Second, we examined the weak invariance model or metric invariance. In this model, the factor loadings are treated as invariant across gender. This ensures that the measures are considered to be on the same scale across gender for making valid comparisons. Third, we examined the strong invariance model. This model imposes invariance on both factor loadings and item intercept across gender. This is to ensure the underlying factors can be compared across gender. Fourth, we examined the strict invariance model, which requires the factor loadings, intercepts, and residual variances to be invariant. This is to examine whether the variances of the regression equations for each item are invariant across gender.

Once the measurement invariance was established, we investigated the invariance of structural parameters. First, we examined the invariance of factor variance and factor covariance to determine whether the relationships between underlying factors remained unchanged in different genders. Then we examined the factor mean invariance to test the factor mean differences between male and female samples. Non-invariance of the structural parameters does not indicate a problem with the measure being studied; it indicates heterogeneity among the comparison groups (Wang and Wang, 2012).

Evidence of invariance between the less restrictive model (e.g., configural invariance model) and more restrictive model (e.g., weak measurement invariance models) were based on recommendations from the literature (Cheung and Rensvold, 2002; Chen, 2007; Wang and Wang, 2012; Kimber et al., 2015). A value of the change in CFI (ΔCFI) smaller than or equal to 0.01 indicates that the hypothesis of invariance should not be rejected. For ΔTLI and ΔRMSEA, the critical values are 0.01 and 0.015, respectively. The Chi-square difference test was also reported for each comparison.

## Results

### Descriptive Statistics for the Sample by Gender

The main physical and leisure activities reported by the 13- to 17-year-old males and females in the sample included jogging, cycling, badminton, basketball, taekwondo, netball, and wushu. **Table 1** shows means and SDs for demographic variables and the seven motives for participating in physical activity by gender. Males reported a larger number of physical activity sessions per week than females, and their sessions were also longer than those of females, indicating that males undertook considerably more physical activity than females during a typical week.

**Table 1:** Means and standard deviations (SD) for demographic variables and motives for participation in physical activity by gender.

Variables	Males (*n* = 334) Mean (*SD*)	Females (*n* = 449) Mean (*SD*)	*t*-statistics (df = 781)	*p*-value
Age, years	14.62 (1.41)	14.42 (1.13)	1.94	0.053
Number of session of physical activity per week	2.88 (1.94)	2.10 (1.31)	6.70	<0.001
Duration per session in minutes	65.26 (55.25)	48.55 (45.68)	4.63	<0.001
Motives for participating in physical activity:				
Enjoyment	3.96 (0.69)	3.79 (0.72)	3.33	<0.001
Mastery	3.96 (0.65)	3.76 (0.65)	4.67	<0.001
Affiliation	3.71 (0.72)	3.48 (0.73)	4.30	<0.001
Appearance	4.15 (0.68)	4.18 (0.65)	-0.66	0.509
Competition/ego	3.41 (0.79)	3.06 (0.72)	6.60	<0.001
Physical condition	3.96 (0.73)	3.75 (0.72)	3.93	<0.001
xsPsychological condition	3.91 (0.72)	3.90 (0.75)	0.43	0.669

### Measurement Model PALMS-Y-M

The hypothesized measurement model (hypothesized model) for PALMS-Y-M for the whole sample, including both genders, consists of seven factors with 28 items, which is four items in each factor. The test of this model (Model-1) did not result in a good fit to the data (CFI = 0.905). Model-1 was reestimated after each respecification based on MI and with adequate empirical support. These modifications of Model-1 included adding residual covariances on items Q1 with Q2, Q9 with Q10, and Q10 with Q15. The final respecified model (Model-2) fit the data well based on several fit indices (CFI = 0.924, RMSEA = 0.046, SRMR = 0.048).

Baseline models for males (Model-4) and females (Model-6), after respecification, reflect a satisfactorily good fit to the data based on several fit indices (Model-4: CFI = 0.922, RMSEA = 0.048, SRMR = 0.050; Model 6: CFI = 0.922, RMSEA = 0.047, SRMR = 0.053). Similar, but not completely identical, baseline models were identified for males and females. Baseline models for males and females included adding two residual covariances (items 1 and 2, items 6 and 8) and six residual covariances (items 1 and 2, items 10 and 15, items 9 and 10, items 22 and 27, items 19 and 21, items 6 and 11), respectively. The standardized factor loadings for each factor within the male and female models are illustrated in **Table 2**.

**Table 2:** Standardized factor loadings for final models.

Factors	Items	Standardized factor loading, all samples^a^	Standardized factor loading	
			Male sample^b^	Female sample^c^
Enjoyment	Q1	0.564	0.497	0.607
	Q9	0.712	0.745	0.702
	Q18	0.779	0.776	0.783
	Q25	0.679	0.666	0.672
Mastery	Q2	0.658	0.619	0.681
	Q12	0.716	0.758	0.682
	Q17	0.674	0.671	0.670
	Q23	0.661	0.690	0.602
Competition/ego	Q3	0.568	0.560	0.505
	Q13	0.735	0.752	0.776
	Q19	0.708	0.753	0.589
	Q21	0.654	0.680	0.515
Affiliation	Q4	0.626	0.651	0.634
	Q14	0.708	0.727	0.719
	Q22	0.535	0.526	0.405
	Q27	0.710	0.666	0.676
Appearance	Q6	0.744	0.679	0.790
	Q8	0.769	0.702	0.794
	Q11	0.781	0.824	0.783
	Q20	0.756	0.735	0.770
Physical condition	Q7	0.586	0.639	0.523
	Q16	0.740	0.777	0.715
	Q24	0.692	0.691	0.685
	Q28	0.787	0.737	0.825
Psychological condition	Q5	0.639	0.617	0.652
	Q10	0.738	0.741	0.741
	Q15	0.738	0.806	0.689
	Q26	0.774	0.779	0.772

*^a^Adding residual covariance between item 1 and 2, 9 and 10, and 10 and 15.^b^Adding residual covariance between items 1and 2 and 6 and 8. ^c^Adding residual covariance between items 1 and 2, 10 and 15, 9 and 10, 22 and 27, 19 and 21, and 6 and 11*.

### Factor Correlational Structure

**Table 3** details the correlational structure of the seven factors of the PALMS-Y-M based on standardized covariance values for males and females. The standardized covariance values for males ranged from 0.52 to 0.93 and for females they ranged from 0.39 to 0.92. High standardized covariance values between some factors were expected. For example, latent factor covariances between Enjoyment and Mastery were high (0.92) for both males and females because these two latent factors both reflect the higher-order factor of intrinsic motivation, which is consistent with previous study using the “parent” scale, REMM (Rogers, 2000, unpublished).

**Table 3:** Standardized factors covariance estimated for males and females.

Latent factors	1	2	3	4	5	6	7
1. Enjoyment	–	0.92	0.63	0.76	0.87	0.75	0.85
2. Mastery	0.92	–	0.75	0.69	0.93	0.89	0.89
3. Competition/ego	0.59	0.74	–	0.74	0.52	0.70	0.56
4. Affiliation	0.59	0.57	0.44	–	0.59	0.60	0.63
5. Appearance	0.79	0.83	0.46	0.40	–	0.87	0.84
6. Physical condition	0.65	0.68	0.41	0.39	0.66	–	0.78
7. Psychological condition	0.84	0.80	0.41	0.47	0.85	0.61	–

*Top diagonal = males, bottom diagonal = females. All covariances are significant at p < 0.001.*

### Measurement and Structural Invariance

The two baseline models had the same seven factors, and we found that all 28 items fell into their hypothesized factors (motives). These two baseline models were then integrated into the configural invariance model, with the same number of factors and the same pattern of fixed and free factor loadings, but no equality restrictions were imposed on any parameter across genders. The configural invariance model fit the data well (see **Table 4**). This configural model was then used to compare against the more restrictive measurement invariance (i.e., weak measurement invariance) model that we examined next.

**Table 4:** Malay version of the Physical Activity and Leisure Motivation Scale for Youth (PALMS-Y-M) baseline model fit results and tests of measurement invariance.

Models	MLR_x_^2^ (df)	CFI	TLI	RMSEA	SRMR	Model comparison	ΔMLR_x_^2∗^ (df), *p*-value	ΔCFI	ΔTLI	ΔRMSEA
Model-1 (all samples: hypothesized)	992.605 (329)	0.905	0.891	0.051	0.050	–	–	–	–	–
Model-2^a^ (all samples: respecified)	856.130 (326)	0.924	0.912	0.046	0.048	–	–	–	–	–
Model-3 (male group: hypothesized)	620.943 (329)	0.908	0.894	0.052	0.052	–	–	–	–	–
Model-4^b^ (male group: respecified)	574.131 (327)	0.922	0.910	0.048	0.050	–	–	–	–	–
Model-5 (female group: hypothesized)	800.757 (329)	0.885	0.868	0.057	0.061	–	–	–	–	–
Model-6^c^ (female group: respecified)	643.973 (323)	0.922	0.908	0.047	0.053	–	–	–	–	–
Model-7 (configural)	1215.713 (650)	0.922	0.909	0.047	0.052	–	–	–	–	–
Measurement invariance		
Model-8 (weak)	1252.149 (671)	0.920	0.909	0.047	0.057	8 versus 7	35.484 (21), 0.025	-0.002	0	0
Model-9 (strong)	1305.029 (692)	0.915	0.907	0.048	0.057	9 versus 8	57.193 (21), <0.001	-0.005	0.002	0.001
Model-10 (strict)	1360.722 (720)	0.911	0.907	0.048	0.062	10 versus 9	55.137 (28), 0.002	-0.004	0	0
Structural invariance									
Model-11 (factor variance and covariance)	1356.137 (720)	0.912	0.908	0.048	0.066	11 versus 9	51.251 (28), 0.005	-0.003	0.001	0
Model-12 (factor variance, covariance and factor mean)	1414.497 (727)	0.905	0.901	0.049	0.070	12 versus 11	78.485 (7), < 0.001	-0.007	-0.007	0.001

*^∗^Corrected value. ^a^Adding residual covariance between item 1 and 2, 9 and 10, and 10 and 15. ^b^Adding residual covarianc ebetween items 1 and 2 and 6 and 8. ^c^Adding residual covariance between items 1and 2, 10 and 15, 9 and 10, 22 and 27, 19 and 21, and 6 and 11. Δ = change in value. MLR, robust to maximum likelihood; df, degree of freedom; CFI, comparative fit index; TLI, Tucker and Lewis index; RMSEA, root mean square error of approximation; SRMR, standardized root mean residual*.

The first more restrictive model, the weak invariance model, fit the data well (see **Table 4**). Changes of CFI, TLI, and RMSEA, when the weak invariance model is compared with the configural invariance model, were within acceptable values (ΔCFI = −0.002, ΔTLI = 0, ΔRMSEA = 0). This indicates that the metric of factor scores was invariant across gender. In other words, the items used to estimate the factor loadings have the same meaning for males and females. The next restrictive model, the strong invariance model also fit the data well (see **Table 4**). The second more restrictive model, which constrained the factor loadings and item intercept to create the strong invariance model, resulted in the demonstration of strong invariance (ΔCFI = −0.005, ΔTLI = 0.002, ΔRMSEA = 0.001). This indicates that both factor loadings and item intercept are invariant between genders. The last more restrictive model, which constrained the factor loadings, item intercept, and residual variances, to produce the strict invariance model was then inspected. The changes of the fit indices were within the recommended values (ΔCFI = −0.004, ΔTLI = 0, ΔRMSEA = 0). This suggests that the average item score comparisons are valid between males and females.

Further invariance testing on structural parameters revealed that the factor variance and covariances of the model of PALMS-Y-M were invariant between males and females in this sample. The structural invariance for the factor variance and covariances model fit the data well based in RMSEA (0.048) and SRMR (0.062). The differences of several fit indices with the less restrictive invariance model (i.e., strong measurement invariance model) are within the acceptable values (ΔCFI = −0.003, ΔTLI = 0.001, ΔRMSEA = 0). In other words, the same relationships between the seven factors measured by the PALMS-Y-M remain among the two genders. Factor mean invariance (Model-12) was also tested to see whether the mean of each factor was different among male and female samples. Model-12 fit the data well based on RMSEA (0.049) and SRMR (0.070). When comparing with the less restrictive model (i.e., factor variance and covariance), the differences of several fit indices are within the acceptable values (ΔCFI = −0.007, ΔTLI = −0.007, ΔRMSEA = 0.001).

## Discussion

The development of the PALMS-Y is an important step in determining individuals’ participation in physical activity among adolescents. Among the questionnaires that measure motives for participation in physical activity, PALMS measures a wider range of motives than most other questionnaires. PALMS measures motives across recreational and lifestyle physical activity, as well as competitive sport (Zach et al., 2012). PALMS-Y is a shortened version of PALMS that specifically measures the motives for participation in physical and leisure activity among youth. The Malay language translation of this 28-item measure, PALMS-Y-M, which was used in the present study, confirmed the multidimensionality of the motives for participation in physical activity. Overall, the seven-factor model of PALMS-Y-M showed an acceptable fit (CFI = 0.924, RMSEA = 0.046, SRMR = 0.048). This is consistent with the previous study on the PALMS-M, where we found that the validity of the adult measure was acceptable based on several fit indices (Kueh et al., 2017a).

The present study provides new insight on measurement invariances of PALMS-Y-M across gender. We tested the measurement and structural invariances of PALMS-Y-M among a youth sample in Kota Bharu, Malaysia. Exploration on the first two levels revealed metric or factor loading invariance (i.e., weak measurement invariance) and scalar invariance (i.e., strong measurement invariance) of the seven-factor model across gender. Metric invariance is important to ensure the measure across multiple groups is considered to be on the same scale, or the factors are measured in the same way in all groups (Vandenberg and Lance, 2000; Meredith and Teresi, 2006; Wang and Wang, 2012). Scalar invariance refers to the item intercept being invariant across multiple groups in the present study. This indicates that none of the groups tends to respond systemtically higher or lower to the items of scales than other groups (Vandenberg and Lance, 2000; Meredith and Teresi, 2006; Wang and Wang, 2012). The present study met both invariance requirements. These results confirm that the two youth samples, male and female, had an equivalent understanding on each of the 28 items in the measure, which is an important prerequisite for making a meaningful comparison between gender on motives for participation in physical and leisure activity.

Researchers have argued that error variance invariance (i.e., strict measurement invariance) is not required for substantive analyses in many disciplines and such invariance is considered unnecessary (Wang and Wang, 2012). However, error variance invariance is crucial when difference of items’ reliability across groups is of concern. This is because error variance invariance is considered as invariance of item reliabilities across groups (Schmitt et al., 1984), given that the factor variances are invariant across groups (Vandenberg and Lance, 2000). The present finding on PALMS-Y-M measured across gender met the strict measurement invariance criterion and exceeded the psychometric requirements in invariance testing. Further, invariance tests on the structure of PALMS-Y-M provided convincing results on the invariance of factor variance and covariance. These indicated that the relationships between the seven motives (factors) under study remain unchanged between males and females. The psychometric findings were favorable all along the line with measurement and structural invariance testings suggested in the literature (Meredith and Teresi, 2006; Wang and Wang, 2012). This study also yielded additional information on factor means invariance across gender. According to Wang and Wang (2012), we need to ensure that the scale operates equivalently across comparison groups, but we do not expect that the levels of latent variables remain unchanged among different groups. However, the present study has indicated homogeneity of the mean factors among male and female samples based on the structural invariance for factor variance, covariance, and factor mean.

In the present study, we decided to add covariance between residuals’ items within the same factor, as well as from different factors. These modifications on the hypothesized PALMS-Y-M model were decided based on the MI values reported in Mplus output and after adequate theoretical support was gathered by the researchers. Two residual covariances involved items from different factors. These were item Q1 [because it’s interesting (Factor: Enjoyment)] with Q2 [to get better at an activity (Factor: Mastery)], and item Q9 [because it makes me happy (Factor: Enjoyment)] with Q10 [to get away from pressures (Factor: Psychological Condition)]. Covariance between residuals for items Q1 and Q2 was reasonable, although both items are from different factors. This is because the Mastery and Enjoyment factors are two components of the higher-order factor intrinsic motivation based on previous study (Rogers, 2000, unpublished). Thus, when individuals want to improve their skill in an activity, it is likely that reflects an interest in the activity. Conversely, when individuals are interested in a skill-based activity, it is likely they will want to improve their skill in that activity. These two items could be linked together due to their common background in Intrinsic Motivation. Similarly, with covariance between residuals for items Q9 and Q10, if one removes pressures or escapes them, one should certainly feel happier. Conversely, feeling happy is incompatible with experiencing pressure. Other residuals’ covariances added in the baseline models for males and females were within their latent factors. The residuals’ covariances for the male sample were items within the factor Appearance (Q6: because it helps maintain a healthy body and Q8: be physically fit) and for the female sample were items within the factor Psychological condition (Q10: to get away from pressures and Q15: because it acts as a stress release), factor Affiliation (Q22: to talk with friends exercising and Q27: to be with friends), factor Competition/ego (Q19: to work harder than others and Q21: to compete with others around me), and factor Appearance (Q6: because it helps maintain a healthy body and Q11: to maintain physical health). These residual covariances were added in the models after consideration of substantive meaningfulness, in order to achieve a better fitting model. This is not suprising. Especially in social psychological research, these parameters can make strong substantive sense, thus, they should be included in the model (Jöreskog and Sörbom, 1993; Cole et al., 2007).

Estimated values of latent factor covariances reflect on their discriminability and, thus, on the extent of the factors dissimilar in PALMS-Y-M. In the present study, some high factor covariances between latent factors are worth noting and discussing. Based on the previous study on the “parent” scale, REMM was created incorporating both theory-based and atheoretical approaches (Rogers, 2000, Unpublished). Rogers’ (2000, Unpublished) study was a qualitative study that involved in-depth, semi-structured interviews with exercise participants, to examine their reasons for participation in non-competitive physical activity. The study fit neatly into the framework of self-determination theory (SDT; Deci and Ryan, 1985, 1991), namely intrinsic-extrinsic motivation. In the present study, both the male and female samples indicated high factor covariance between Enjoyment and Mastery. This is expected because both factors reflect intrinsic motivation as reported in Rogers’ (2000, Unpublished) study, which fits the theoretical framework of SDT. In addition, strict requirement of zero cross-loadings in CFA or fixing items’ factor loading to be zero usually leads to over-estimated factor correlations (Asparouhov and Muthén, 2009). This may also explain the high correlation between latent variables observed in the present study. However, based on previous studies, discriminant validity was found to be satisfactory with correlations between the eight motive factors below the acceptable value of 0.85 (Kline, 2011) for the Malay version of REMM (Kueh et al., 2017b) and PALMS (Kueh et al., 2017a) regardless of gender. We further examined the correlation between factors of PALMS-Y-M in the present study. Based on standardized covariance values, regardless of gender (i.e., combined samples), all values were below 0.85 except for Enjoyment and Mastery (0.92). Although one may propose to combine the factors, which were highly correlated, doing so may cause discrepancy in interpretation of the motives’ score in PALMS-Y-M among researchers in the future. Therefore, we proposed that the framework of seven motives of PALMS-Y-M should continue to be interpreted as seven separate domains of motives in adolescent, male and female, samples.

These findings have practical implications for researchers, teachers, physical educators, and health planners who are interested in using the PALMS-Y-M to assess motives for participation in physical and leisure activity in youth. This information can guide practitioners to advise adolescents about the kinds of activities that would satisfy their primary motives, which should lead to long-term participation. Future research needs to be carried out to examine the stability of PALMS-Y and PALMS-Y-M across times by conducting longitudinal studies. Longitudinal CFA models can be applied to examine the stability of cross-time relations of PALMS-Y-M motives and whether the motives change over time. This approach can be extended to the study of factors that influence change by using longitudinal models in the presence of systematic interventions designed to enhance specific motives. In addition, Malaysia is a multicultural country with different types of ethnic groups, including Malay, Chinese, Indian, and the aboriginal. Thus, it would be interesting to examine measurement invariance of PALMS-Y-M among different cultures to ensure the measure is equivalent among different cultural groups in Malaysia.

We acknowledge that self-report survey data is subject to response bias, which may decrease the accuracy of the data provided by the participants. Besides, based on impression management processes, participants may answer the questions so that their responses would make them look good. We bore these response biases in mind during data collection in the present study. Thus, we constantly encouraged and reminded the participants to respond honestly to all questions related to their motives for participation of physical and leisure activities. Also, we assured them of confidentiality, stressing that their answers would not be seen by their teachers, and their responses would not have any effect on their academic performance.

## Conclusion

The present study provided evidence of the validity of PALMS-Y-M and suggests that the measure demonstrated measurement invariance (i.e., configural, weak, strong, and strict) and structural invariance (i.e., factor variance, covariance, and mean). These findings provided new information that the PALMS-Y-M items were perceived similarly between males and females in this sample from Kota Bharu, Malaysia. Future research on motives for participation among youth can employ the PALMS-Y-M to examine motives for engaging in any form of physical activity and leisure activities, interpreting their responses within the seven-factor framework of subscales, and making meaningful comparisons across gender.

## Author Contributions

YK, GK, and NA developed the concept and design of the study. YK, GK, and NA conducted the study, participated in data collection, analyzed, and interpreted the data. YK and NA drafted the original manuscript. TM and NN provided critical revisions to the manuscript. All authors read and approved the final manuscript.

## Conflict of Interest Statement

The authors declare that the research was conducted in the absence of any commercial or financial relationships that could be construed as a potential conflict of interest.

## Appendix 1

### The Physical Activity and Leisure Motivation Scale for Youth (PALMS-Y)

In responding to the following statements, think of the motives you have for the physical activity you do. Try not to spend time pondering over your responses. There are no right or wrong answers. Indicate how much your motives correspond with each of the statements. In each case **1** indicates **strongly disagree** and **5** indicates **strongly agree**.

**Table A1:**

	I undertake physical activity.....	Strongly disagree	Disagree	Neutral	Agree	Strongly agree
1	because it’s interesting	1	2	3	4	5
2	to get better at an activity	1	2	3	4	5
3	because I perform better than others	1	2	3	4	5
4	to do activity with others	1	2	3	4	5
5	to better cope with stress	1	2	3	4	5
6	because it helps maintain a healthy body	1	2	3	4	5
7	to define muscle, look better	1	2	3	4	5
8	be physically fit	1	2	3	4	5
9	because it makes me happy	1	2	3	4	5
10	to get away from pressures	1	2	3	4	5
11	to maintain physical health	1	2	3	4	5
12	to improve existing skills	1	2	3	4	5
13	to be best in the group	1	2	3	4	5
14	to do something in common with friends	1	2	3	4	5
15	because it acts as a stress release	1	2	3	4	5
16	to improve body shape	1	2	3	4	5
17	to obtain new skills/activities	1	2	3	4	5
18	because it’s fun	1	2	3	4	5
19	to work harder than others	1	2	3	4	5
20	because it keeps me healthy	1	2	3	4	5
21	to compete with others around me	1	2	3	4	5
22	to talk with friends exercising	1	2	3	4	5
23	to keep current skill level	1	2	3	4	5
24	to improve appearance	1	2	3	4	5
25	because I enjoy exercising	1	2	3	4	5
26	to take my mind off other things	1	2	3	4	5
27	to be with friends	1	2	3	4	5
28	to maintain a trim, toned body	1	2	3	4	5

### The Malay Version of Physical Activity and Leisure Motivation Scale for Youth (PALMS-Y-M)

#### Skala Motivasi Aktiviti Fizikal dan Masa Lapang untuk Belia (PALMS-Y-M)

Dalam memberi respons kepada kenyataan berikut, fikirkan apa motif anda untuk melakukan aktiviti fizikal yang selalu anda pilih untuk lakukan. Cuba untuk tidak menghabiskan masa yang banyak memikirkan jawapan anda. Tidak ada jawapan yang betul atau salah. Nyatakan sejauh mana motif anda bersetuju dengan setiap kenyataan. Dalam setiap kes, (1) menunjukkan sangat tidak setuju, dan (5) menunjukkan sangat setuju.

**Table A2:**

	Saya melibatkan diri di dalam aktiviti fizikal…	Sangat tidak setuju	Tidak setuju	Neutral	Setuju	Sangat setuju
1	kerana ia adalah menarik	1	2	3	4	5
2	untuk menjalankan aktiviti dengan lebih bagus	1	2	3	4	5
3	kerana saya melakukan sesuatu dengan lebih baik berbanding orang lain	1	2	3	4	5
4	untuk melakukan aktiviti bersama-sama dengan orang lain	1	2	3	4	5
5	untuk menangani tekanan dengan lebih baik	1	2	3	4	5
6	kerana ia membantu saya mengekalkan badan yang sihat.	1	2	3	4	5
7	supaya otot kelihatan lebih bagus	1	2	3	4	5
8	untuk menjadi cergas	1	2	3	4	5
9	kerana ia membantu saya berasa gembira	1	2	3	4	5
10	untuk menghilangkan tekanan	1	2	3	4	5
11	untuk mengekalkan kesihatan fizikal	1	2	3	4	5
12	untuk meningkatkan kemahiran yang sedia ada	1	2	3	4	5
13	untuk menjadi yang terbaik dalam kumpulan.	1	2	3	4	5
14	Untuk melakukan sesuatu yang sama dengan rakan-rakan	1	2	3	4	5
15	kerana ia mampu melegakan tekanan	1	2	3	4	5
16	untuk memperbaiki bentuk badan saya.	1	2	3	4	5
17	untuk mendapatkan kemahiran yang baru atau aktiviti baru	1	2	3	4	5
18	kerana ia adalah menyeronokkan	1	2	3	4	5
19	untuk bekerja dengan lebih kuat daripada orang lain	1	2	3	4	5
20	kerana ia dapat mengekalkan kesihatan saya	1	2	3	4	5
21	untuk bersaing dengan orang lain	1	2	3	4	5
22	untuk berbual dengan rakan-rakan semasa bersenam	1	2	3	4	5
23	untuk mengekalkan tahap kemahiran semasa saya	1	2	3	4	5
24	untuk memperbaiki penampilan saya	1	2	3	4	5
25	kerana saya suka bersenam	1	2	3	4	5
26	untuk menenangkan fikiran saya daripada perkara lain	1	2	3	4	5
27	untuk bersama-sama dengan rakan-rakan saya	1	2	3	4	5
28	untuk mengekalkan badan yang langsing dan tegap	1	2	3	4	5

#### Scoring

For each motive subscale sum the four scores to produce a total score for that subscale between 4 and 20 and keep the subscales as continuous measures of the seven motives to participate in physical activity and leisure.

Enjoyment items: 1, 9, 18, 25 Mastery items: 2, 12, 17, 23 Competition/ego items: 3, 13, 19, 21 Affiliation items: 4, 14, 22, 27 Appearance items: 6, 8, 11, 20 Physical condition items: 7, 16, 24, 28 Psychological condition items: 5, 10, 15, 26
